# c.A2456C-substitution in *Pck1* changes the enzyme kinetic and functional properties modifying fat distribution in pigs

**DOI:** 10.1038/srep19617

**Published:** 2016-01-21

**Authors:** Pedro Latorre, Carmen Burgos, Jorge Hidalgo, Luis Varona, José Alberto Carrodeguas, Pascual López-Buesa

**Affiliations:** 1 Departamento de Producción Animal y Ciencia de los Alimentos, Facultad de Veterinaria, Universidad de Zaragoza, 50013 Zaragoza, Spain; 2 Instituto de Biocomputación y Física de Sistemas Complejos, Universidad de Zaragoza, 50018 Zaragoza, Spain; 3 Departamento de Anatomía, Embriología y Genética, Facultad de Veterinaria, Universidad de Zaragoza, 50013 Zaragoza, Spain; 4 Departamento de Bioquímica y Biología Molecular y Celular, Facultad de Ciencias, Universidad de Zaragoza, 50009 Zaragoza, Spain; 5 IIS Aragón, 50009 Zaragoza, Spain

## Abstract

Cytosolic phosphoenolpyruvate carboxykinase, PCK1, is one of the main regulatory enzymes of gluconeogenesis and glyceroneogenesis. The substitution of a single amino acid (Met139Leu) in PCK1 as a consequence of a single nucleotide polymorphism (SNP), c.A2456C, is associated in the pig to a negative phenotype characterized by reduced intramuscular fat content, enhanced backfat thickness and lower meat quality. The p.139L enzyme shows reduced *k*_*cat*_ values in the glyceroneogenic direction and enhanced ones in the anaplerotic direction. Accordingly, the expression of the p.139L isoform results in about 30% lower glucose and 9% lower lipid production in cell cultures. Moreover, the ability of this isoform to be acetylated is also compromised, what would increase its susceptibility to be degraded *in vivo* by the ubiquitin-proteasome system. The high frequency of the c.2456C allele in modern pig breeds implies that the benefits of including c.A2456C SNP in selection programs could be considerable.

Pig selection based just on control of phenotypic traits has allowed important changes in pig and meat properties in the last decades. Selection performed during this time has been directed to obtain lean carcasses and pigs with efficient feed conversion; since the sixties feed conversion indexes have been reduced from 4.3 down to 2.2 and backfat thickness (BT) from about 3 cm down to a few mm^1^,^2^. The instrument to achieve this goal has been the measurement of backfat thickness due to the strong negative genetic correlation (−0.70 to −0.90) between ultrasound measurement of BT in living animals and their carcass lean content^3^ and also due to the easiness of its *in vivo* measurement using ultrasonic echography. However, the reduction in BT has resulted also in a reduction of intramuscular fat content (IMF) from about 3% down to 1%^1^,^2^ because there exists a positive genetic correlation of up to 0.60 between these two traits^3^. Current pigs are very efficient biological machines converting feed into meat, but the problem with their meat is precisely that it suffers from a lack of intramuscular fat; present pigs yield meat of low sensorial quality because many meat quality traits such as taste, juiciness or flavor are strongly dependent on intramuscular fat content^4^,^5^. Selecting pigs for simultaneous carcass leanness and high intramuscular fat content is not easy due to the mentioned positive genetic correlation between both traits and also because the measurement of intramuscular fat *in vivo* is far from being straightforward.

Pig breeding in the last decades has also been facilitated by marker-assisted selection; marker-assisted selection consists in the use of gene markers associated to the desired meat or carcass properties or, much better, genes with a direct effect on meat or carcass traits. Many of these gene markers are single nucleotide polymorphisms (SNPs). The most important SNP used in pig breeding, *RYR1* c.C1843T, the halothane gene, has a strong influence on both meat quality and carcass conformation^6^. Other important SNPs for pig breeding are located within the *IGF2* locus^7^,^8^, a growth factor that has strong effects on muscularity, or in *MC4R*^9^, which is involved in regulating feeding behavior, modifying in this way the carcass and meat fat content. All these gene markers were discovered in the pig after their biological roles or effects, especially on weight and adiposity gain, were described in mice and/or in human beings. For example, *RYR1* involvement in malignant hyperthermia in humans^10^ fueled research in the pig gene and led to the discovery of the causal mutation of pig malignant hyperthermia and of the related appearance of pale, soft and exudative (PSE) pork. The finding of mutations in porcine *MC4R* was prompted by the discovery of its role in obesity in humans and mice^11^,^12^ and the investigation of the pig *IGF2* gene was initiated after its involvement in human and mouse development had been described^13^,^14^. Clearly, comparative biology has demonstrated its usefulness for pig breeding.

Finding SNPs that could simultaneously increase intramuscular fat, to improve meat quality, and reduce BT, to improve the economy of pig production, is not easy due to the already mentioned positive genetic correlation between both traits, but it is not impossible. Indeed, an *IGF2* SNP has been shown to strongly decrease BT and, slightly, but significantly, increase IMF^15^. Markers such as that of *IGF2* are very advantageous for both breeders and consumers and their finding can have considerable economic value. In the search for more SNPs able to simultaneously reduce BT and to increase IMF content we have directed our attention to comparative biology: in 2007 a transgenic mouse overexpressing 100 fold the cytosolic form of phosphoenolpyruvate carboxykinase (Pck1) in skeletal muscle was shown to have almost four fold larger intramuscular fat content (IMF) and strongly reduced amounts of both subcutaneous and visceral fat^16^. Because this is exactly the desirable phenotype in pig breeding schemes we thought that the *Pck1* pig gene could be a good candidate to search for variability.

*Pck1* is also an interesting candidate gene due to its involvement in lipid and carbohydrate metabolism. PCK1 catalyzes the conversion of oxaloacetate to phosphoenolpyruvate with concomitant GTP hydrolysis to yield GDP^17^. It is situated in a crossroad of metabolic pathways: it plays a regulatory role in gluconeogenesis, in serine biosynthesis, and in anaplerosis and cataplerosis of the tricarboxylic acid (TCA) cycle^18^. In addition, and especially in tissues where no gluconeogenesis occurs, it plays a fundamental role in glyceroneogenesis, providing glycerol-3-phosphate as a precursor for fatty acid esterification in triglyceride synthesis^19^. Changes in Pck1 activity have been reported by several authors to result in changes in the amount and distribution of body fat in several species^20^,^21^,^22^,^23^; these results reinforce the potential of *Pck1* as a candidate gene to search for polymorphisms that could cause changes in fat content and/or distribution in pigs.

To study the potential role of Pck1 in fat deposition and distribution in the pig we have sequenced the whole promoter and coding regions of the *Pck1* gene in breeds or crosses (Iberian, Piètrain, Duroc x Landrace/Large White) that differ amply in fat-related traits. In this analysis we have found a SNP in the coding sequence of *Pck1* that results in a methionine to leucine substitution. This SNP is strongly associated to changes in fat content and distribution and also on meat quality in the Duroc x Landrace/Large White cross; the analysis of the effects of the SNP on kinetic, structural and functional properties of purified Pck1 also reveals significant differences between Pck1 isoenzymes, reinforcing the hypothesis that c.A2456C contributes to the phenotypic changes detected in the Duroc x Landrace/Large White cross.

## Results

### *Pck1* gene sequencing and analysis

We have used a set of five pairs of primers to amplify and sequence the whole coding region of the *Pck1* gene including all introns, with some gaps. The analyzed sequence excludes the initial 5´UTR-region in exon one that is not translated into protein. The promoter region was amplified with another set of four pairs of primers.

**Figure 1:**
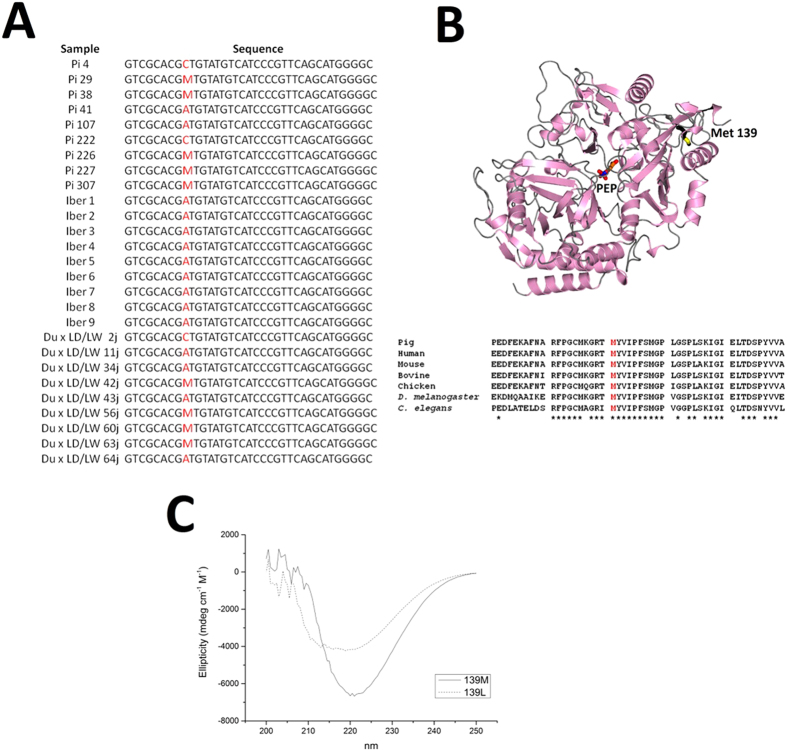
c.A2456C substitution in pig *Pck1* and its consequences on protein CD-spectra. **(A)** Sequences from 27 pigs ranging from nucleotide 2448 to 2483 show the substitution at position 2456. Nucleotide 2456 (red) from exon 4, which followed a different segregation pattern between the three pig breeds (Pi = Piètrain, Iber = Iberian and Du x LD/LW = Duroc x Landrace/Large White), produced a methionine to leucine substitution at position 139 of the protein. A = Adenine, C = Cytosine, G = Guanine, T = Thymine and M = Heterozygous (A/C). **(B)** Crystal structure of human PCK1 (PDB accession 1KHF) showing that Met139 is far away from the active site, represented here by bound PEP. Alignment of the PCK1 protein region containing Met139 shows this amino acid is conserved from man to *C. elegans*. Asterisks indicate perfectly conserved amino acids among species sequences obtained from GenBank (pig, NP_001116630; human, NP_002582; mouse, NP_035174; bovine, NP_777162; chicken, NP_990802; Drosophila, NP_001097367 and *C. elegans*, NP_001021589). **(C)** CD spectra between 190 and 250 nm were recorded for each isoenzyme using three different preparations at a concentration of 20 μM and 25 °C. The figure shows a representative image of simple spectra for both proteins. Spectra from both isoenzymes are clearly different.

No single SNP was found in the promoter region that segregates differentially between Iberian and Piètrain pigs. However, we have found one SNP, c.A2456C (see Fig. 1A), in exon 4 of Piètrain pigs whose allelic frequency (0.5) was statistically different (p < 0.05) to that of Iberian pigs. The substitution segregates also in the Du x LD/LW cross. The SNP results in an amino acid change, a methionine to leucine substitution at position 139 of the protein. The substituted amino acid is located in a β-strand far away from the active site of the enzyme (Fig. 1B) but in a region of about 20 amino acids that is highly conserved from man to *C. elegans* (Fig. 1B).

### Association study between c.A2456C and phenotypic traits in the Du x LD/LW cross

We analyzed the c.A2456C substitution in the *Pck1* gene in 202 animals from the same Du x LD/LW cross to evaluate its effects on fat distribution and meat quality traits. The analysis was made using an RT-PCR assay performed on a previously amplified DNA fragment containing the c.A2456C substitution (see suppl. Figure 1). The genotypic frequencies in this cross were 0.361 AA, 0.565 AC and 0.074 CC. We performed a Bayesian analysis of association between the two alleles and the phenotypic traits altered in the *Pck1* transgenic mouse (BF and IMF) and also with some meat quality traits. The results are shown in Table 1. The A allele is clearly associated to larger (up to 20.4%) IMF content in two of the three analyzed muscles (Bayesian posterior probability above cero ranging from 0.9973 in *L. dorsi* to 0.9800 in *Ps. major*) and also to 9.9% lower BT (Bayesian posterior probability above cero 0.0409), showing also a similar, although not as extreme, pattern to the transgenic *Pck1* mouse. In addition, a strong association was found between higher water holding capacity at several time points postmortem and the A allele (Bayesian posterior probability above cero ranging from 0.0174 to 0.0404). This elevated (up to 24%) water holding capacity is probably related to the higher values of pH_24_ also associated to A alleles (see Table 1).

### Kinetic properties of Pck1p.139Met and Pck1 p.139Leu isoenzymes

In order to demonstrate if the detected polymorphism in Pck1 had an effect on the catalytic properties of the enzyme, what would reinforce the hypothesis that c.A2456C is contributory and not merely in linkage disequilibrium with the potential causative mutation(s), we purified and characterized kinetically both isoenzymes as His-tagged proteins (suppl. Figure 2). His-tagged Pck1 has been shown to behave almost identically to the non His-tagged enzyme^24^. We calculated apparent values of *K*_m_, *k*_cat_ and *k*_cat_/*K*_m_ for the five Pck1 substrates (see suppl. Figure 3 and Table 2).

Pck1 p.139Met showed significantly larger *k*_cat_ (between 35% and 60% larger) values for the two substrates (OAA and GTP) in the direction of PEP synthesis, the pathway that leads ultimately to the synthesis of glycerol phosphate precursors for triglyceride synthesis. Pck1 p.139Met showed also significantly lower *k*_cat_ values (about 50% lower) for two substrates (PEP and GDP) in the reverse reaction. That means that Pck1 p.139Leu is able to catalyze this reverse reaction at relatively higher speed than Pck1 p.139Met. There is no clear trend for *K*_m_ values: Pck1 p.139Met shows significant lower *K*_m_ values for PEP and GDP but also significant larger *K*_m_ values for KHCO_3_. There is also no clear trend in *k*_cat_/*K*_m_ values in the direction of OAA synthesis; Pck1 p.139Leu shows significant larger values of this parameter for KHCO_3_ whereas it exhibits significant lower ones for GDP. No significant differences have been found in *k*_cat_/*K*_m_ values of PEP. We have not found any differences in *k*_cat_/*K*_m_ values for substrates in the direction of PEP synthesis.

### Physicochemical characterization of Pck1

In order to detect structural changes caused by the amino acid substitution we followed different approaches. First, we determined the CD spectra of both isoenzymes. As seen in Fig. 1C these spectra were quite different, indicating that there are differences in the secondary structure between both isoenzymes. Quantification of β-sheet and α-helix content was not possible because of the low resolution of the apparatus below 200 nm.

Second, we calculated D_t_ values at 50 °C in the absence or presence of substrates (see Table 3 and suppl. Figure 4). We found significant differences between Pck1 p.139Met and Pck1 p.139Leu heat resistance in the absence of substrate; the former was about 1.3 fold more heat resistant than the latter. When substrates were added to the heating medium, PEP was the substrate that conferred most protection to both isoenzymes although the difference between heat resistance of Pck1 p.139Leu and Pck1p.139Met were low. OAA represents the other extreme; it does not protect any isoenzyme against heat inactivation. Largest differences in heat resistance between isoenzymes were found when the nucleotide substrate, either GDP or GTP, was present in the heating medium: both enhanced more than 2 fold the heat resistance of only Pck1 p.139Met having no effect on Pck1 p.139Leu. As GTP and GDP bind to the same residues in the active center of the enzyme, these results indicate that the conformational change in the surrounding of the nucleotide-binding site due to the SNP, located far apart from the former, is large enough to change the heat resistance of the enzyme when the nucleotide is present.

Third, we have compared the susceptibility of both isoforms to proteolytic degradation using trypsin or proteinase K, in independent experiments. The analysis of protein fragments by SDS-PAGE showed no difference (see suppl. Figure 5).

The attempts to crystallize both isoforms to determine their three dimensional structure have failed so far although we have tried to obtain crystals under about 600 different conditions.

### Effects of Pck1 overexpression on glucose production in cell cultures

**Figure 2:**
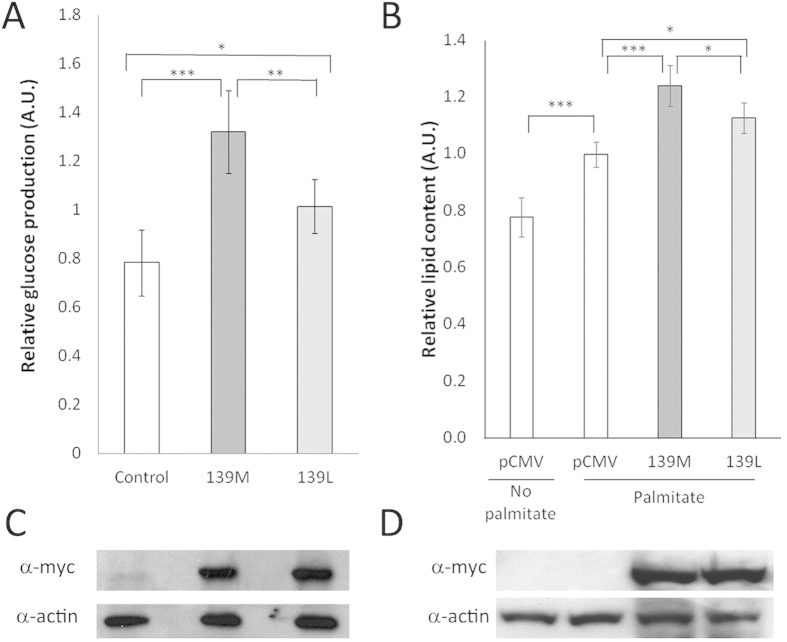
Effect of *Myc-Pck1* overexpression on glucose and lipid production in HEK293 cells. **(A)** Relative glucose production of cells transfected using an empty pCMV-Myc plasmid (0.78 ± 0.13) as control and those expressing Myc-Pck1 p.139Met (139M; 1.32 ± 0.17) or Myc-Pck1 p.139Leu (139L; 1.01 ± 0.11) using DMEM without glucose and phenol red and supplemented with 2 mM sodium pyruvate and 20 mM sodium lactate. Glucose was determined using a colorimetric assay (GAGO20, Sigma). Shapiro-Wilk test was performed to check data normality. One-way ANOVA (n = 4) shows differences between groups (p = 0.000128). A post hoc Tukey test was performed to obtain differences between groups (*p < 0.05, **p < 0.01, ***p < 0.001). **(C)** Western blot to analyze Pck1 p.139Met and Pck1 p.139Leu expression levels using an anti-myc antibody. Actin was included as loading control. **(B)** Relative lipid production of cells transfected using an empty pCMV-Myc plasmid without palmitate (0.78 ± 0.07) or with palmitate (1 ± 0.04) as controls and those expressing Myc-Pck1 p.139Met (139M; 1.24 ± 0.07) or Myc-Pck1 p.139Leu (139L; 1.13 ± 0.05) in the presence of palmitate, using complete DMEM. Lipids were determined by Oil Red staining and normalized to the protein content from the whole cell lysates. Shapiro-Wilk test was performed to check data normality. One-way ANOVA (n = 3) shows differences among groups (p = 2.16 × 10^−06^). A post hoc Tukey test was performed to obtain differences between groups (*p < 0.05, **p < 0.01, ***p < 0.001). **(D)** Western blot to show myc-Pck1 levels of each protein isoform in the lipid determination assays.

Pck1 is one of the main regulators of gluconeogenesis^25^. We have studied the effects of Pck1 p.139Leu and Pck1 p.139Met overexpression on the gluconeogenic capacity of HEK293 cells. Pck1 isoenzymes were independently overexpressed in HEK293 cells and the production of glucose was measured after 24 hours. Expression levels of both isoenzymes were similar (see Fig. 2B) as analyzed by western blotting but the amount of glucose produced was significantly larger, around 30%, in cells expressing Pck1 p.139Met than in cells expressing Pck1 p.139Leu (see Fig. 2A).

### Effects of Pck1 overexpression on lipogenesis in cell cultures

Since the glyceroneogenic pathway can ultimately lead to triglyceride synthesis, we determined accumulation of lipids in cultured cells using a colorimetric assay based on binding of Oil Red to lipids. We compared HEK293 cells transfected with empty vector with those overexpressing either Pck1 isoform, treated with palmitic acid to stimulate lipogenesis. Results, shown in Fig. 2, panels C and D, indicated a significant (9%) increase in lipids in cells overexpressing Pck1 p.139Met with respect to those overexpressing Pck1 p.139Leu.

### Acetylation of Pck1 p.139Leu and Pck1 p.139Met in cell cultures

**Figure 3:**
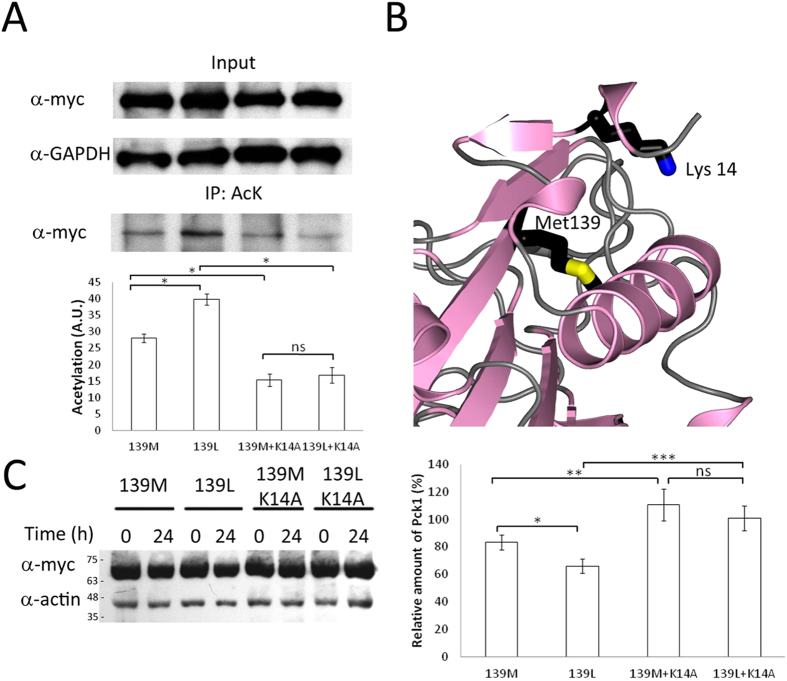
Acetylation susceptibility of Pck1 p.139Met and Pck1 p.139Leu. **(A)** Immunoprecipitation assays (40 μg of protein each) were performed using an anti-acetyl-lysine (AcK) antibody and detected with an anti-myc antibody by western blot. GAPDH was used as loading control. Results show differences between Pck1 p.139Met (139M) and Pck1 p.139Leu (139L) that disappeared when Lys14 was replaced with alanine in both proteins (139M+K14A and 139L+K14A). The illustration shows a 20 second exposure for the input and a 60 second exposure for immunoprecipitates. Western blots were analyzed using ImageJ. The difference in acetylation between Pck1 p.139Met and Pck1 p.139Leu (n = 6) was statistically significant (p = 1.38 × 10^−15^). Since data did not meet with normality a Kruskal-Wallis test was performed and a Wilcoxon rank sum test was used to obtain differences between groups (*p < 0.05, ns = not significant). A.U., arbitrary units. **(B)** Crystal structure of human PCK1 (PDB accession 1KHG) showing that Lys14 is relatively close (8.9 Å) to Met139. **(C)** Pck1 p.139Met and p.139Leu levels in HEK293 cells treated with 100 μg/mL cycloheximide (CHX) for 24 hours. Pck1 p.139Met is less sensitive to degradation than Pck1 p.139Leu (20%). When Lys14 is replaced with alanine both isoenzymes increased its stability up to 100%. Values shown are relative to the amount of Pck1 at time 0 of the assay, before cycloheximide addition, and have been normalized to the amount of actin in each sample. Numbers on the left of the blot indicate the molecular mass of protein standards run in parallel. Data shown in the graph represent three individual experiments with two technical replicates each. Statistical analyses were carried out as indicated in the legend to Fig. 2, showing differences among groups (p = 0.000685) and between groups (*p < 0.05, **p < 0.01).

Acetylation has been proposed to be an important regulator of Pck1 activity^26^. Acetylation is a label to direct Pck1 to proteolytic degradation by the ubiquitin-proteasome system. Pck1 is acetylated in at least three lysine residues^25^. We have analyzed the acetylation degree of Pck1 p.139Leu and Pck1 p.139Met expressed in HEK293 cells using antibodies specific for acetylated lysine residues. As shown in Fig. 3A, Pck1 p.139Leu exhibits significantly larger (32%) acetylation than Pck1 p.139Met. Expression levels of Pck1 isoenzymes in these experiments were also similar as measured by western blotting.

The difference in acetylation degree between Pck1 p.139Leu and Pck1 p.139Met disappeared when the lysine at position 14 was substituted by alanine (see Fig. 3A). This lysine is the one which lies closer (about 9 Ångstroms) to residue 139 in the three dimensional structure of the enzyme (see Fig. 3B). These results suggest that Pck1 p.139Leu is a better substrate for acetylation and, therefore, that it probably has a lower proteolytic stability than Pck1 p.139Met *in vivo*.

### Protein stability in cellular cultures

To study if differential protein acetylation influenced protein stability, HEK293 cells overexpressing either Pck1 c.139Met or Pck1 c.139Leu were treated with cycloheximide (CHX) to inhibit protein synthesis. This allows following the stability of already synthesized Pck1. Pck1 was quantified by western blotting using anti-myc 24 hours after CHX addition. Actin was used as loading control. Pck1 p.139Met amount (mean ± SD) was 81 ± 4.8% (n = 3) whereas Pck1 p.139Leu was only 63 ± 5.5% (see Fig. 3C). This difference was statistically significant (p = 0.013). When Lys14 was substituted by Ala in both Pck1 p.139Met and Pck1 p.139Leu PCK1, protein levels did not change for 24 hours after CHX addition showing the relevance of Lys14 to regulate Pck1 stability.

### Allelic frequencies of *Pck1* in diverse breeds or crosses

Table 3 shows that the *Pck1* c.2456C allele, the one that encodes leucine, is present in many breeds or crosses and even in wild pigs although at substantial different frequencies. The *Pck1* c.2456A allele, the one that encodes methionine and is associated to both better meat quality and more favorable fat distribution, seems to be overrepresented in breeds or crosses not subjected to strong artificial selection (Duroc breed 1 is an ancient Duroc population preserved in Spain that has not been artificially selected since the fifties) whereas the *Pck1* c.2456C allele appears at higher frequencies in breeds, such as the Piètrain, that have been selected for high lean content.

## Discussion

We describe in this work how the observation that a 100-fold overexpression of Pck1 in mouse skeletal muscle led to the identification of a polymorphism in Pck1 associated to an increase in fat deposition in pig skeletal muscle together with a decrease in subcutaneous fat, the same phenotype observed in the mouse.

We found a single SNP, c.A2456C, that segregated differently in Piètrain (low intramuscular fat and thin adipose cover) and Iberian pigs (high intramuscular fat and thick adipose cover), and caused an amino acid change: a methionine instead of a leucine at position 139 of the protein (Met139Leu). The SNP segregated also in 9 pigs of the Du x LD/LW cross (intermediate phenotype). The substitution is relatively conservative since both leucine and methionine belong to the aliphatic nonpolar amino acid group, although methionine, having an S atom in its lateral chain, is more mobile. Despite the substitution being located far away from the active center of the protein, in a region of about 20 amino acids highly conserved from *C. elegans* to man, our results indicate that it is responsible for catalytic differences in this enzyme. These long-range effects are at the basis of allosteric phenomena in proteins, which had not been described in Pck1 until the very recent report by Balan and coworkers^27^.

Our genotypic analyses of a group of pigs of the Du x LD/LW cross for which we had phenotypic data related to fat and meat quality indicated that the A allele (Met) is strongly associated to the desired phenotype, i.e., higher intramuscular fat and thinner adipose cover. The A allele showed also a strong association to larger water retention capacity, one of the most important meat quality traits, which is probably related to the higher pH_24_ values also associated to this allele^28^.

To determine whether these differences are caused by differences in Pck1 activity and not just to linkage disequilibrium with the real causal polymorphisms, we analyzed the activities *in vitro* of both recombinant isoenzymes and found that they were, kinetically, very different: Pck1 p.139Met is more active (k_cat_ values 35-60% higher) in the direction of PEP synthesis, the glyceroneogenic pathway that can ultimately lead to triglyceride synthesis, whereas it is less active in the opposite direction (k_cat_ values 40-50% lower), that of OAA synthesis. The opposite occurs with Pck1 p.139Leu. These differences in activities in either direction of the reaction are remarkable as the enzyme can function *in vivo* in both directions^18^. We therefore observe in the pig the same phenomenon than Hakimi *et al.*^16^ observed in the muscles of mice: more glyceroneogenic Pck1 activity is associated to larger intramuscular fat content.

The increased *in vitro k*_*cat*_ of Pck1 p.139Met in the cataplerotic direction of the reaction, that is, in the glyceroneogenic and gluconeogenic direction, is probably at the origin of the differences in glucose production and lipid accumulation in cells overexpressing this isoenzyme compared with cells that overexpress Pck1 p.139Leu. Our results using cultured cells overexpressing either isoform show that the kinetic differences observed *in vitro* can have a direct translation to more complex biological systems.

Kinetic differences between both isoenzymes should be the consequence of structural changes in the protein, and our analyses of the CD-spectra and thermal stability of both isoenzymes indicated that this is indeed the case. Both isoenzymes differ clearly in their CD-spectra, i.e, in the relative abundance of α-helices and β-sheets, and also differ considerably in resistance to heat inactivation, which is a consequence of changes in enzyme structure. Very subtle changes in Pck1 protein structure have been shown to have large effects on its resistance to heat inactivation^29^. Moreover, the addition of substrates to the heating medium had a very different stabilization effect on the two isoenzymes, since nucleotides (GDP and GTP) stabilize Pck1 p.139Met but not Pck1 p.139Leu. This points to the existence of structural differences in the close vicinity of the nucleotide-binding site between both isoenzymes. Accordingly, the largest significant differences in kinetic parameters are those found for GDP and GTP. The fact that we have not observed differences in the susceptibility of both isoenzymes to proteolytic degradation can be very likely due to lack of significant conformational differences in exposed regions or regions of the protein that lack secondary structure, which are the regions preferentially affected by proteases.

Since changes in acetylation of Pck1 affect its proteolytic stability^26^, with acetylated protein being directed to the proteasome for degradation, we tested whether Pck1 p. 139Met and Pck1 p.139Leu showed different acetylation levels, and this was in fact the case. Pck1 p.139Leu showed higher acetylation in cell culture, which was related to a lower stability. Furthermore, substitution of lysine 14, which lies in the three-dimensional structure close (9 Ångstroms) to residue 139 (see Fig. 3), by alanine abolished these differences, increasing enzyme stability to near 100%. The effects of Met139Leu substitution on total Pck1 activity are therefore at least due to a double mechanism: a change in *k*_cat_ and a decrease in proteolytic stability due to increased acetylation.

She *et al.*^22^ insightfully noted that a change in activity levels of Pck1, an enzyme located in the intersection of several fundamental metabolic pathways such as gluconeogenesis and glyceroneogenesis and involved in anaplerotic^18^ or cataplerotic^30^ regulation of Krebs cycle, could yield the most unexpected results. There seems to be a correlation between the expression level of *PCK1* in a particular tissue and triglyceride accumulation in that tissue^20^,^21^,^22^,^23^, being perhaps the mouse overexpressing Pck1 in muscular tissue^16^ the most spectacular example. However, a *Pck1* knockout mouse^31^ showed the apparent paradoxical decrease of total body fat content together with increased fat content in the liver. High levels of Pck1 activity in muscles have been also shown to correlate with a lean phenotype in rats^32^. In addition, a change in expression or activity of PCK1 in one tissue, and the metabolic changes associated to it, have been shown to strongly influence Pck1 expression in other tissues^16^. Our results represent nevertheless a different scenario, demonstrating that expression in all tissues where Pck1 is expressed of different isoforms of the enzyme can have different effects in fat accumulation in different tissues.

Transgenic mice overexpressing Pck1 in muscle^16^ show lowered blood lactate levels, with lower glycogen degradation and glycolysis, since their energy metabolism relies mainly on oxidative phosphorylation, with their muscles having more mitochondria. Although we have not analyzed these parameters in our pigs, the higher pH_24_ value associated to the *Pck1* c.2456A allele suggests that the extent of glycogen degradation and of glycolysis are also lower in pigs carrying that allele.

The central position of Pck1 in lipid and carbohydrate metabolism made it a clear candidate to modulate several important phenotypic traits. Recent results showing that Pck1 expression in pig muscles correlates with IMF content^33^ reinforce the idea of the involvement of Pck1 in fat synthesis in pig muscles. The opposite effects of c.A2456C on backfat and IMF traits facilitates the selection of these traits independently and efficiently even despite their positive genetic correlation^3^. The fact that the A allele is associated to larger intramuscular fat content and also to higher water retention capacity makes it a very interesting marker to be used if meat quality is a main selection goal. Selecting any allele of *PCK1* would not be difficult because the allelic frequencies in diverse breeds (Table 4) show that the SNP segregates in all of them.

Apart from the importance of the polymorphisms reported in this work for pig selection, pigs carrying either isoenzyme could also be very useful to gain insight into the multiple roles of PCK1 in global metabolism^18^, obesity^23^, diabetes^34^ or cancer^35^ due to the differences in their kinetic and regulatory properties, as well as models to study the effects of acetylation on enzyme activity and stability^36^. Our work, which initially had an unequivocal applied goal, could therefore be interesting for research in fundamental metabolic questions proving that sometimes basic research can benefit from applied one.

## Methods

### Animal material

Animals used in this study were raised in an experimental farm but were treated as regular production animals in every sense (feeding, transport or slaughtering) in accordance with the approved guidelines.

DNA for sequencing *Pck1* was obtained from semen of Piètrain pigs from Agropecuaria Obanos SA (Marcilla, Spain), from muscles of both pure Iberian pigs, supplied by Dr. Carmen García (Universidad de Extremadura, Spain), and of a Duroc x Landrace/Large White cross from PORTESA (Teruel, Spain).

Muscle samples for DNA extraction for SNP genotyping of Duroc pigs were supplied by Censyra (Badajoz, Spain), PORTESA (Teruel, Spain), and Dr. Noelia Ibáñez-Escriche (IRTA, Lérida, Spain); those of wild pigs were supplied by several hunting friends of our Departments; those of pure Iberian pigs were supplied by Dr. Carmen García (Universidad de Extremadura, Spain). Semen or hair follicles were used to extract DNA for genotyping Piètrain and Piètrain x Large White pigs, respectively; both pig populations were from Agropecuaria Obanos SA (Marcilla, Spain).

The association study between the c.A2456C substitution and several phenotypic traits was performed with 202 pigs of both sexes (the males were castrated) from 9 sires and 32 dams of a Duroc x Landrace/Large White cross that were raised in an experimental farm, fed *ad libitum* a standard diet. The pigs were slaughtered after stunning with CO_2_ at an average weight of 114.5+/−10.98 kg.

### Phenotypic recording

Backfat thickness was measured at L5 (lumbar vertebra 5) level with a caliper shortly after slaughtering. pH_45_ (pH 45 minutes postmortem) and pH_24_ (pH 24 hours postmortem) were measured in *L. dorsi* at L2 level with a PC3000 Oakton pHmeter equipped with a Hamilton penetration electrode. Drip loss was measured in quadruplicate in *L. dorsi* samples taken between L2 and T14 (thoracic vertebra 14) as described by Honikel^37^. Fat content was analyzed from 10 g samples taken form *L. dorsi* at T12 level, and calculated gravimetrically from a 5 ml aliquot of the chlorophormic phase. Lipids were extracted by the method of Bligh & Dyer^38^ as modified by Hanson & Olley^39^.

### Cloning and sequencing of pig *Pck1*

#### DNA extraction and quantification

DNA was isolated from 27 pig hair follicle samples using the Real Pure genomic DNA extraction kit (Durviz) and quantified spectrophotometrically.

#### DNA Sequencing

The *Pck1* coding region was amplified by PCR using AccuPrime^TM^ *Taq* DNA Polymerase High Fidelity (Invitrogen) in the presence of 7.5% DMSO. PCR products were purified using NucleoSpin^®^ Extract II kit (Macherey-Nagel) and sequenced. Sequence analyses were carried out using Vector NTI (Life Technologies Corporation) and ClustalW (www.ch.embnet.org/software/ClustalW.html).

#### Pck1 cloning

Total cDNA was obtained using the Cells to cDNA kit (Ambion, USA). *Pck1* cDNA was amplified with AccuPrime^TM^ *Taq* DNA Polymerase High Fidelity and 12.5% DMSO, followed by nested PCR including 5% DMSO. NdeI-XhoI digestion products were cloned into pET15b (provided with a PreScission Protease cleavage site, courtesy of Dr. Ramón Hurtado Guerrero, BIFI, Spain) and EcoRI-XhoI fragments were cloned into pCMV-Myc. DNA was isolated from *E. coli* DH5α colonies and sequenced.

### Site-directed mutagenesis

Site directed mutagenesis was performed on pCMV-Myc clones in order to substitute lysine by alanine at position 14 using the QuickChange Site-Directed mutagenesis Kit (Stratagene) with Pfu Ultra II HS fusion DNA polymerase (Agilent). Mutated DNA was sequenced to confirm the desired changes.

### Genotyping

Detection of polymorphism at position 2456 of the *Pck1* gene was performed by Real Time (RT)-PCR using a DNA fragment previously amplified by standard PCR, since RT-PCR on genomic DNA failed to detect the c.A2456C substitution. The experimental approach was designed using Assay by Design (Applied Biosystems) with appropriate Taqman probes labeled with VIC or FAM fluorochromes.

### Protein expression and purification

Proteins were overexpressed in *E. coli* BL21 (DE3) by induction with 1 mM IPTG at 37 °C followed by analyses by SDS-PAGE. Since part of the desired protein was present in the pellet after centrifugation, lower induction temperatures were used for large-scale preparations.

Large-scale expression was carried out in 2 L of LB medium containing ampicillin at 18 °C for 24 hours. Bacteria were harvested by centrifugation and resuspended in lysis buffer (50 mM sodium phosphate, pH 7.4, 500 mM NaCl, 10 mM imidazole, 2 mM β-mercaptoethanol, 0.1% benzonase (Novagen), 1 mg lysozyme (Sigma), 1 μM PMSF, 10 μM benzamidine and 0.5 μM leupeptin), incubated for 20 minutes at 37 °C and sonicated with a Vibra-Cell sonicator (Sonics & Materials). The lysate was clarified by centrifugation, the pellet discarded and the supernatant was filtered through a 0.45 μm filter and used in further purification steps.

Proteins were purified by affinity chromatography on a 5 ml FF crude HiTrap Talon Column (GE Healthcare Life Sciences), using an UPC-900 (GE Healthcare Life Sciences) HPLC apparatus with UNICORN Manager software. The column was first equilibrated with buffer A (50 mM NaH_2_PO_4_, pH 7.4, 500 mM NaCl, 10 mM imidazole, 2 mM β-mercaptoethanol). After sample loading, the column was washed with buffer A and bound protein was eluted with a linear gradient from 100% buffer A to 100% buffer B (same as buffer A but with 300 mM imidazole). Fractions were collected, analyzed by SDS-PAGE and those containing Pck1 were pooled and concentrated using Amicon® Ultra Centrifugal Filters 30 kDa (Millipore). A final buffer exchange was performed with a HiPrep 26/10 column (GE Healthcare Life Sciences) equilibrated in buffer C (20 mM HEPES, pH 7.4, 150 mM NaCl, 1 mM TCEP). After chromatography, Pck1 was concentrated again with Amicon® Ultra Centrifugal Filters 30kDa and aliquots were flash-frozen in liquid nitrogen and stored at −80 °C.

For structural assays, Pck1 was treated with PreScission Protease (GE Healthcare) to eliminate the His-tag and then loaded onto a HisTrap column equilibrated in buffer D (20 mM HEPES, pH 7.4, 500 mM NaCl, 10 mM imidazole and 1 mM TCEP). Pck1 was collected in the flow-through, concentrated and loaded onto a HiLoad Superdex 75 column equilibrated in buffer C. Fraction purity was evaluated by SDS-PAGE and pure fractions were concentrated and loaded onto a HiPrep 26/10 desalting column equilibrated in buffer E (20 mM HEPES, pH 7.4, 1 mM TCEP). Protein concentration was calculated by absorbance determination at 280 nm and by Bradford assays.

### Enzyme assays and kinetic calculations

Pck1 activity was measured with coupled spectrophotometric assays in both reaction directions^40^ using an UNICAM 500 spectrophotometer. Assays in the direction of oxaloacetic acid synthesis were performed in 1 ml of a 100 mM HEPES buffer, pH 7.2, containing 10 mM DTT, 0.2 mM MnCl_2_, 2 mM MgCl_2_, 2 mM GDP, 0.2 mM NADH, 2 mM PEP, 100 mM KHCO_3_ and 2 units of malate dehydrogenase. Assays were initiated by addition of 4 μg of Pck1. Rates were calculated by measuring the decrease in absorbance at 340 nm after subtracting the rate of spontaneous NADH oxidation. Assays in the direction of phosphoenolpyruvate synthesis were performed in 1 ml of a 100 mM HEPES buffer, pH 7.2, containing 10 mM DTT, 0.2 mM MnCl_2_, 2 mM MgCl_2_, 1 mM GTP, 1 mM ADP, 0.2 mM NADH, 1 μg of Pck1, and 5 units each of pyruvate kinase and lactate dehydrogenase. Reactions were initiated by addition of oxaloacetate (OAA). Rates were calculated by measuring the decrease in absorbance at 340 nm after subtracting the rate of the blank which contained all the components of the mix but Pck1. Apparent values of *K*_*m*_, *k*_*cat*_ and *K*_*m*_*/k*_*cat*_ were calculated in triplicate in independent sets of assays using a non-linear regression software^41^. In these assays, saturating substrate concentrations (2 mM PEP, 2 mM GDP, 100 mM KHCO_3_, 1 mM GTP or 400 μM OAA) were used except for the variable substrate.

### Circular dichroism (CD) analysis of purified Pck1

Pck1 was diluted to 20 μM in buffer E. Readings were performed at 25 °C in a 0.1 cm cuvette from 190 to 250 nm in a Chirascan CD-spectrophotometer (Applied Photophysics). This experiment was repeated three times with different protein samples.

### Heat resistance determinations

D_t_ values, the time (in minutes) required to reduce the initial activity to 1/10 of the original, were determined by measuring residual enzymatic activities after heating 20 μL enzyme aliquots inside 100 μL capacity capillary tubes (Drummond Scientific Co) for different times at constant temperature (50 °C). Capillary tubes were immediately cooled down in an ice bath and the residual enzymatic activity immediately measured. Residual activity in experiments with no added substrates or in the presence of PEP or GDP was measured in the direction of OAA synthesis at saturating substrate concentrations and in the PEP-synthesis direction in the presence of OAA or GTP, also at saturating substrate concentrations. At least 6 different time points were used for each D_t_ value calculation. R^2^ was over 0.9 in all these calculations. D_t_ values were calculated in triplicate by linear regression. We show average values of these three calculations.

### Sensitivity to proteolytic cleavage

Purified Pck1 samples were subjected to proteolytic degradation using trypsin (Sigma) or Proteinase K (Durviz) at 39 °C; aliquots were taken after different time points, mixed with standard SDS buffer, boiled for 5 minutes and analyzed by SDS-PAGE. Band quantification was performed with a densitometer “Imagemaster D Platinum 7” and software Image Scanner III (GE Healthcare Life Sciences).

### Glucose assays in cell cultures

We followed the same approach described in previous works^26^,^42^. HEK293 cells (5 × 10^5^) were grown on 6-well plates in 3 mL of DMEM supplemented with 10% fetal bovine serum, 2 mM L-glutamine, 0.1 mg/mL streptomycin, and 100 U/mL penicillin. Cells were incubated at 37 °C, 5% CO_2_ until reaching ca. 80% confluence. Then, cells were transfected using GeneJuice reagent (Novagen). 24 hours after transfection, the medium was replaced with 1 mL of DMEM without glucose and phenol red, and supplemented with sodium pyruvate and sodium lactate to a final concentration of 2 and 20 mM, respectively. After 3 hours, half of the medium was collected and glucose concentration was measured with a colorimetric assay (GAGO20 from Sigma). Data was normalized to the total protein content of cell lysates. Cells transfected with empty pCMV-Myc were used as control. Pck1 expression was analyzed by western blot from whole cell lysates using anti-myc (Invitrogen) and anti-actin (Sigma) in 50 mM Tris-HCl, pH 7.5, 150 NaCl, 5% BSA.

### Lipid assays in cell cultures

1.25 × 10^5^ HEK293 cells were grown on 24-well plates, previously treated with poly-L-lysine, for 24 h in complete DMEM as indicated above. Cells were transfected using Genejuice and 24 hours later the medium was replaced with complete DMEM supplemented with 250 μM palmitic acid (Sigma), previously dissolved in DMSO at 75 mM. After 24 hours cells were fixed in 3.7% formaldehyde in 1 × PBS for 1 h at room temperature. Cells were washed twice with distilled water and treated with 60% isopropanol for 5 minutes. Dry cells were stained with Oil Red O (Sigma, 0.2% in 60% isopropanol) for 30 minutes with shaking and washed four times with distilled water to eliminate unbound oil red. Then, bound Oil Red was dissolved in 1 mL of 100% isopropanol for 1 hour with shaking. Relative lipid content was determined by measuring Oil Red absorbance at 500 nm and normalized to the protein content from the whole cell lysates. This experiment was repeated three times with three technical replicates each.

### Analysis of Pck1 acetylation in HEK293 cells

HEK293 cells were transfected as previously described. 24 hours after transfection, DMEM was replaced with 400 μL of cell lysis buffer (1xPBS, pH 7.4, 0.5% Tween 20, 1 mM EDTA, 0.1 mM EGTA, 1 μM PMSF, 10 μM benzamidine, 10 μM trichostatin A and 0.5 μM leupeptin). Cells were incubated on ice for 15 minutes and collected by centrifugation. 40 μg of protein (Bradford assays) were immunoprecipitated overnight with anti acetyl-lysine antibody (Cell Signal) which was then bound to Dynabeads Protein G (Invitrogen) for 3 hours at 4 °C. Immunoprecipitates were extensively washed using 1xPBS, 0.5% Tween 20 (PBST), resuspended in SDS-loading buffer, boiled and analyzed by western blot following SDS-PAGE. Anti-myc (Invitrogen) and anti-GAPDH Genetex) primary antibodies were used followed by incubation with anti-rabbit-HRP and anti-mouse-HRP (Millipore) and detection by ECL (Millipore). Proteins were quantified by densitometric analysis of the films using ImageJ software (NIH).

### Analysis of the effect of lysine acetylation on protein stability in cell culture

HEK293 cells (1.25 × 10^5^) were seeded on 24-well plates and transfected as previously described. 24 h after transfection, the medium was replaced with fresh DMEM containing cycloheximide (CHX, Sigma) at 100 μg/mL to stop protein synthesis. Cells were harvested in SDS-lysis buffer after 24 h, boiled and protein decay was analyzed by western blotting using ImageJ software comparing Pck1 levels just after CHX addition and after 24 hours.

### Statistical analysis

Data for the association study were analyzed for each trait separately by using a Bayesian approach (equation 1). The assumed Bayesian likelihood was:


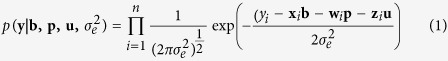


where **y** is the vector of phenotypic records for the analyzed trait, **b** is the vector of systematic effects, including covariates with age and the substitution effect of the analyzed polymorphism, a batch effect with four levels and a sex effect with two levels, **p** is the vector of litter effects and **u** represents the polygenic effects. Further, **x**_**i**_, **w**_**i**_ and **z**_**i**_ are the rows of the incidence matrices (**X**, **W** and **Z**) corresponding to the *ith* phenotypic record and 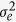 is the residual variance.

Prior distributions for litter and polygenic effects were the following multivariate Gaussian distributions (MVN): 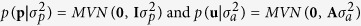, 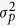 and 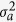 being the litter and polygenic additive variance, respectively and **A** is the numerator relationship matrix. In addition, prior distributions for systematic effects and variance components were assumed uniform with appropriate bounds.

The analysis was performed through a single long chain of 500,000 iterations of a Gibbs Sampler^43^ after discarding the first 25,000 with the TM program^44^. Later on, samples for the substitution effect were used to compute the posterior probabilities above zero.

Shapiro-Wilk normality test was performed before carrying out any other test. Glucose production assay data were analyzed using one-way ANOVA test following a Tukey *post-hoc* test. Acetylation assays did not meet with normality and a Kruskal-Wallis test was performed following a Wilcoxon rank sum test adjusted to Bonferroni method to look for differences between groups. Kinetic parameters, D_t_ values and protein stability in cell culture data were analyzed and compared using two-sample *t*-tests.

## Additional Information

**How to cite this article**: Latorre, P. *et al.* c.A2456C-substitution in *Pck1* changes the enzyme kinetic and functional properties modifying fat distribution in pigs. *Sci. Rep.* **6**, 19617; doi: 10.1038/srep19617 (2016).

## Supplementary Material

Supplementary Information

## Figures and Tables

**Table 1:** Raw mean (and standard deviation-SD-) of meat and carcass traits; posterior mean estimate of the substitution effect (and posterior standard deviation-PSD-) and Bayesian posterior probability above zero (P) of the substitution c.A2456C.

Parameter	Mean (SD)	Substitution effect (PSD)	CC vs. AA difference (%)	P^1^
Backfat thickness	19.88 (5.74)	0.9796 (0.5631)	9.86	0.0409
pH_45_	6.34 (0.23)	0.0028 (0.0292)	0.09	0.4618
pH_24_	5.64 (0.15)	−0.0215 (0.0192)	−0.68	0.8686
Drip loss after 2 days	4.25 (1.89)	0.3821 (0.2189)	17.98	0.0404
Drip loss after 4 days	6.54 (2.37)	0.6065 (0.2874)	18.54	0.0174
Drip loss after 7 days	8.08 (2.47)	0.5819 (0.3130)	14.40	0.0315
% IMF^2^ *L. dorsi*	2.67 (0.95)	−0.2729 (0.0980)	−20.44	0.9973
% IMF^2^ *Ps. major*	1.96 (0.44)	−0.1085 (0.0528)	−11.07	0.9800
% IMF^2^ *B. femoralis*	2.95 (0.87)	0.0040 (0.0992)	0.27	0.4839
% Fat content in adipose tissue	77.78 (5.90)	0.1631 (0.5973)	0.42	0.3924

^1^P = Bayesian posterior probability above zero.

^2^IMF = Intramuscular fat.

**Table 2:** Kinetic properties of Pck1 p.139Met and Pck1 p.139Leu.

Substrate	*K*_m_ (μM)			*k*_cat_ (s^−1^)			*k*_cat_/*K*_m_(M^−1^ s^−1^)		
	139Leu	139Met	Difference^1^,^2^	139Leu	139Met	Difference^1^,^2^	139Leu	139Met	Difference^1^,^2^
PEP	381 ± 53	240 ± 15	1.60**	10.7 ± 0.5	6.8 ± 0.2	1.57**	2.8 × 10^4^	2.9 × 10^4^	0.96
KHCO_3_	7497 ± 513	15560 ± 2163	0.49*	9.2 ± 0.2	10.7 ± 0.5	0.87	1.2 × 10^3^	7.1 × 10^2^	1.70 *
GDP	55 ± 3.6	27.4 ± 2.4	2**	10.7 ± 0.2	7.6 ± 0.2	1.40**	1.9 × 10^5^	2.8 × 10^5^	0.66*
OAA	10.6 ± 0.7	13.1 ± 1.7	0.81	32 ± 0.6	46 ± 1.8	0.69*	3.1 × 10^6^	3.5 × 10^6^	0.88
GTP	23 ± 3.5	45.9 ± 3.8	0.50**	25 ± 1	39 ± 1	0.64**	1.1 × 10^6^	8.8 × 10^5^	1.25

^1^Difference = 139Leu/139Met.

^2^Two-sample *t*-test (n = 3); *p < 0.05, **p < 0.01.

**Table 3:** D_t_ values (min) at 50 °C of Pck1 p.139Met and Pck1 p.139Leu in the absence or presence of different substrates.

	139Met	139Leu	P^1^
No substrate	12.2 ± 0.5	9.3 ± 0.3	**
OAA	12.2 ± 2.1	11.2 ± 4.7	ns
GTP	18.8 ± 0.8	8.8 ± 0.9	***
PEP	89.7 ± 7.6	71 ± 7.8	*
GDP	24.3 ± 0.6	11.8 ± 1.8	***

^1^Two-sample *t*-test (n = 3); *p < 0.05, **p < 0.01, ***p < 0.001, ns: not significant.

**Table 4:** Allelic distribution for the c.A2456C substitution in diverse pig breeds and crosses. p, allelic frequency of the C-allele.

	AA	AC	CC	p (C allele)
Wild pig	10	3	0	0.12
Iberian pig	18	8	0	0.15
Duroc breed line 1	18	8	1	0.19
Duroc breed line 2	17	42	16	0.41
Duroc x Landrace-Large White	73	114	15	0.36
Piètrain x Large White	12	68	53	0.65
Piètrain	2	26	21	0.69

## References

[b1] Haring F. in Handbuch der Tierzüchtung Band 3 (eds. Hammond J., Johansson I. & Haring F.) Ch. 2, 46–100 (Verlag Paul Parey, 1961).

[b2] Birchwood Bulletin. Birchwood Genetics, Inc. (2011). Available at: http://www.birchwoodgenetics.com/files/uploaded/BGI%20Bulletin%20Winter%202011.2012 pdf. (Accessed: 30th April 2015).

[b3] Sellier P. in The Genetics of the Pig (eds. Rothschild M. F. & Ruvinsky A.) Ch. 15, 463–510 (CAB International Publishing, 1998).

[b4] Cannata S. *et al.* Intramuscular fat and sensory properties of pork loin. Ital. J. Anim. Sci. 8 (Suppl. 2), 483–485 (2009).

[b5] Ventanas S., Ruiz J., García C. & Ventanas J. Preference and juiciness of Iberian dry-cured loin as affected by intramuscular fat content, crossbreeding and rearing system. Meat Sci. 77, 324–300 (2007). 22061784 10.1016/j.meatsci.2007.04.001

[b6] Fujii J. *et al.* Identification of a mutation in porcine ryanodine receptor associated with malignant hyperthermia. Science 253, 448–451 (1991). 1862346 10.1126/science.1862346

[b7] Nezer C. *et al.* An imprinted QTL with major effect on muscle mass and fat deposition maps to the IGF2 locus in pigs. Nature Genet. 21, 155–156 (1999). 9988262 10.1038/5935

[b8] Jeon J. T. *et al.* A paternally expressed QTL affecting skeletal and cardiac muscle mass in pigs maps to the IGF2 locus. Nature Genet. 21, 157–158 (1999). 9988263 10.1038/5938

[b9] Kim K. S., Larsen N., Short T., Plastow G. & Rothschild M. F. A missense variant of the porcine melanocortin-4 receptor (MC4R) gene is associated with fatness, growth, and feed intake traits. Mammalian Genome 11, 131–135 (2000). 10656927 10.1007/s003350010025

[b10] MacLennan D. H. *et al.* Ryanodine receptor gene is a candidate for predisposition to malignant hyperthermia. Nature 343, 559–561 (1990). 1967823 10.1038/343559a0

[b11] Yeo G. S. *et al.* A frameshift mutation in MC4R associated with dominantly inherited human obesity. Nature Genet. 20, 111–112 (1998). 9771698 10.1038/2404

[b12] Huszar D. *et al.* Targeted disruption of the melanocortin-4 receptor results in obesity in mice. Cell 88, 131–141 (1997). 9019399 10.1016/s0092-8674(00)81865-6

[b13] Weksberg R., Shen D. R., Fei Y. L., Song Q. L. & Squire J. Disruption of insulin-like growth factor 2 imprinting in Beckwith-Wiedemann syndrome. Nature Genet. 5, 143–150 (1993). 8252039 10.1038/ng1093-143

[b14] DeChiara T. M., Robertson E. J. & Efstratiadis A. Parental imprinting of the mouse insulin-like growth factor II gene. Cell 64, 849–859 (1991). 1997210 10.1016/0092-8674(91)90513-x

[b15] Van Laere A. S. *et al.* A regulatory mutation in IGF2 causes a major QTL effect on muscle growth in the pig. Nature 425, 832–836 (2003). 14574411 10.1038/nature02064

[b16] Hakimi P. *et al.* Overexpression of the cytosolic form of phosphoenolpyruvate carboxykinase (GTP) in skeletal muscle repatterns energy metabolism in the mouse. J. Biol. Chem. 282, 32844–32855 (2007). 17716967 10.1074/jbc.M706127200 PMC4484620

[b17] Utter M. F. & Kurahashi K. Mechanism of action of oxalacetic carboxylase from liver. J. Am. Chem. Soc. 75, 758–787 (1953).

[b18] Yang J., Kalhan S. C. & Hanson R. W. What is the metabolic role of phosphoenolpyruvate carboxykinase? J. Biol. Chem. 284, 27027–27029 (2009). 10.1074/jbc.R109.040543 PMC2785631 19636077

[b19] Hanson R. W. & Reshef L. Glyceroneogenesis revisited. Biochimie 85, 1199–1205 (2003). 14739071 10.1016/j.biochi.2003.10.022

[b20] Olswang Y. *et al.* A mutation in the peroxisome proliferator-activated receptor gamma-binding site in the gene for the cytosolic form of phosphoenolpyruvate carboxykinase reduces adipose tissue size and fat content in mice. Proc. Natl. Acad. Sci. USA 99, 625–630 (2002). 11792850 10.1073/pnas.022616299 PMC117356

[b21] Dong Y. *et al.* A Leu184Val polymorphism in PCK1 gene is associated with type 2 diabetes in Eastern Chinese population with BMI<23 kg/m(2). Diabetes Res. Clin. Pr. 83, 227–232 (2009). 10.1016/j.diabres.2008.10.011 19070910

[b22] She P. *et al.* Phosphoenolpyruvate carboxykinase is necessary for the integration of hepatic energy metabolism. Mol. Cell. Biol. 20, 6508–6517 (2000). 10938127 10.1128/mcb.20.17.6508-6517.2000 PMC86125

[b23] Franckhauser S. *et al.* Increased fatty acid re-esterification by PEPCK overexpression in adipose tissue leads to obesity without insulin resistance. Diabetes 51, 624–630 (2002). 11872659 10.2337/diabetes.51.3.624

[b24] Case C. L. & Mukhopadhyay B. Kinetic characterization of recombinant human cytosolic phosphoenolpyruvate carboxykinase with and without a His(10)-tag. Biochim. Biophys. Acta 1770, 1576–1584 (2007). 17888579 10.1016/j.bbagen.2007.07.012

[b25] Zhao S. *et al.* Regulation of cellular metabolism by protein lysine acetylation. Science 327, 1000–1004 (2010). 20167786 10.1126/science.1179689 PMC3232675

[b26] Jiang W. Q. *et al.* Acetylation Regulates Gluconeogenesis by Promoting PEPCK1 Degradation via Recruiting the UBR5 Ubiquitin Ligase. Mol. Cell 43, 33–44 (2011). 21726808 10.1016/j.molcel.2011.04.028 PMC3962309

[b27] Balan M. D., McLeod M. J., Lotosky W. R., Ghaly M. & Holyoak T. Inhibition and Allosteric Regulation of Monomeric Phosphoenolpyruvate Carboxykinase by 3-Mercaptopicolinic Acid. Biochemistry, 54, 5878-5887 (2015). 26322521 10.1021/acs.biochem.5b00822

[b28] Lawrie R. A. *Ciencia de la carne*, tercera edición. 123-128 Editorial Acribia SA 123-128 (1998).

[b29] Adams D. R. *et al.* Three rare diseases in one Sib pair: RAI1, PCK1, GRIN2B mutations associated with Smith-Magenis Syndrome, cytosolic PEPCK deficiency and NMDA receptor glutamate insensitivity. Mol. Genet. Metab. 113, 161–170 (2014). 24863970 10.1016/j.ymgme.2014.04.001 PMC4219933

[b30] Owen O. E., Kalhan S. C. & Hanson R. W. The key role of anaplerosis and cataplerosis for citric acid cycle function. J. Biol. Chem. 277, 30409–30412 (2002). 12087111 10.1074/jbc.R200006200

[b31] Hakimi P. *et al.* Phosphoenolpyruvate carboxykinase and the critical role of cataplerosis in the control of hepatic metabolism. Nutrition & metabolism 2, 33 (2005). 16300682 10.1186/1743-7075-2-33 PMC1325233

[b32] Novak C. M. *et al.* Endurance capacity, not body size, determines physical activity levels: role of skeletal muscle PEPCK. PloS one 4, e5869 (2009). 19521512 10.1371/journal.pone.0005869 PMC2690400

[b33] Wang W. *et al.* Candidate gene expression affects intramuscular fat content and fatty acid composition in pigs. J. Applied Genet. 54, 113–118 (2013). 23275256 10.1007/s13353-012-0131-z

[b34] Beale E. G., Hammer R. E., Antoine B. & Forest C. Disregulated glyceroneogenesis: PCK1 as a candidate diabetes and obesity gene. Trends Endocrin. Met. 15, 129–135 (2004). 10.1016/j.tem.2004.02.006 15046742

[b35] Zhang P. *et al.* Tumor suppressor p53 cooperates with SIRT6 to regulate gluconeogenesis by promoting FoxO1 nuclear exclusion. Proc. Natl. Acad. Sci. USA 111, 10684–10689 (2014). 25009184 10.1073/pnas.1411026111 PMC4115576

[b36] Tucker A. C., Taylor K. C., Rank K. C., Rayment I. & Escalante-Semerena J. C. Insights into the Specificity of Lysine Acetyltransferases. J. Biol. Chem. 289, 36249–36262 (2014). 25381442 10.1074/jbc.M114.613901 PMC4276886

[b37] Honikel K. O. Reference methods for the assessment of physical characteristics of meat. Meat Sci. 49, 447–457 (1998). 22060626 10.1016/s0309-1740(98)00034-5

[b38] Bligh E. G. & Dyer W. J. A Rapid Method of Total Lipid Extraction and Purification. Can. J. Biochem. Phys. 37, 911–917 (1959). 10.1139/o59-099 13671378

[b39] Hanson S. W. F. & Olley J. Application of the Bligh and Dyer method of lipid extraction to tissue homogenates. Biochem. J. 89, 101–102 (1963).

[b40] Johnson T. A. & Holyoak T. Increasing the conformational entropy of the Omega-loop lid domain in phosphoenolpyruvate carboxykinase impairs catalysis and decreases catalytic fidelity. Biochemistry 49, 5176–5187 (2010). 20476774 10.1021/bi100399e PMC2893389

[b41] Leatherbarrow R. J. ENZFITTER: a non linear regression data analysis program for the IBM-PC. Cambridge: Elsevier Biosoft (1987).

[b42] Yoon J. C. *et al.* Control of hepatic gluconeogenesis through the transcriptional coactivator PGC-1. Nature 413, 131–138 (2001). 11557972 10.1038/35093050

[b43] Gelfand A. E. & Smith A. F. M. Sampling-Based Approaches to Calculating Marginal Densities. J. Am. Stat. Assoc. 85, 398–409 (1990).

[b44] Legarra A., Varona L. & López de Maturana E., *TM Threshold Model* (2008). Available at: http://snp.toulouse.inra.fr/~alegarra/manualtm.pdf. (Accessed: 30th April 2015).

