# Supplementary material for: c.A2456C-substitution in Pck1 changes the enzyme kinetic and functional properties modifying fat distribution in pigs
Source: Sci Rep. 2016 Jan 21;6:19617. doi: 10.1038/srep19617 (PMC4726144; doi:10.1038/srep19617)
Supplement: Supplementary Information [file srep19617-s1.pdf]

SUPPLEMENTARY INFORMATION

c.A2456C-substitution in *Pck1* changes the enzyme kinetic and functional properties modifying fat distribution in pigs

Pedro Latorre[1,2], Carmen Burgos[1,2], Jorge Hidalgo[1], Luis Varona[2,3], José Alberto Carrodeguas*[2,4,5] & Pascual López-Buesa*[1,2]

[1]Departamento de Producción Animal y Ciencia de los Alimentos, Facultad de Veterinaria, Universidad de Zaragoza, 50013 Zaragoza, Spain, [2]Instituto de Biocomputación y Física de Sistemas Complejos, Universidad de Zaragoza, 50018 Zaragoza, Spain, and [3]Departamento de Anatomía, Embriología y Genética, Facultad de Veterinaria, Universidad de Zaragoza, 50013 Zaragoza, Spain. [4] Departamento de Bioquímica y Biología Molecular y Celular, Facultad de Ciencias, Universidad de Zaragoza, 50009 Zaragoza, Spain. [5] IIS Aragón, 50009 Zaragoza, Spain. Correspondence should be addressed to P.L.B. or J.A.C. (e-mail: plopezbu@unizar.es, phone +34 976762533; carrode@unizar.es, phone +34 876555416).

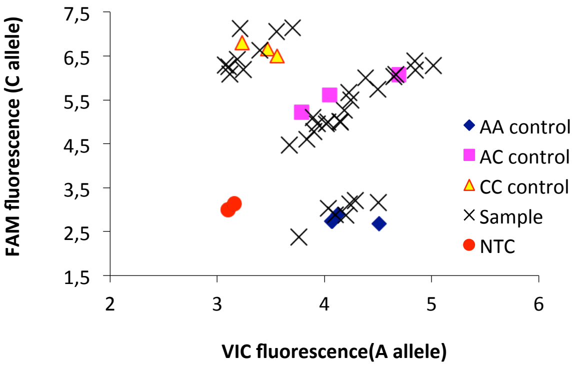

Supplementary Figure 1 | Scatter plot of RT-PCR allelic discrimination assay to genotype *Pck1* c. A2456C polymorphism.

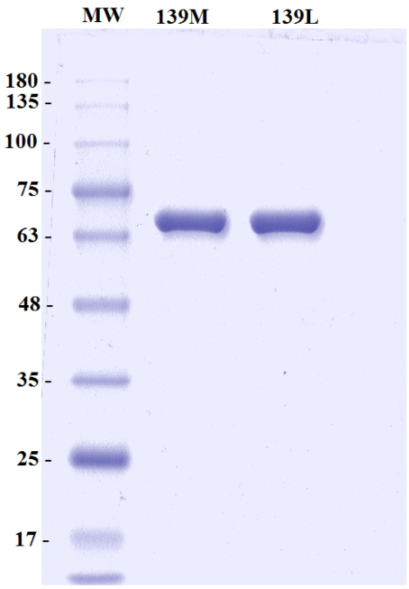

Supplementary Figure 2 I SDS-PAGE of purified Pck1 p.139Met (139M) and Pck1 p.139Leu (139L).

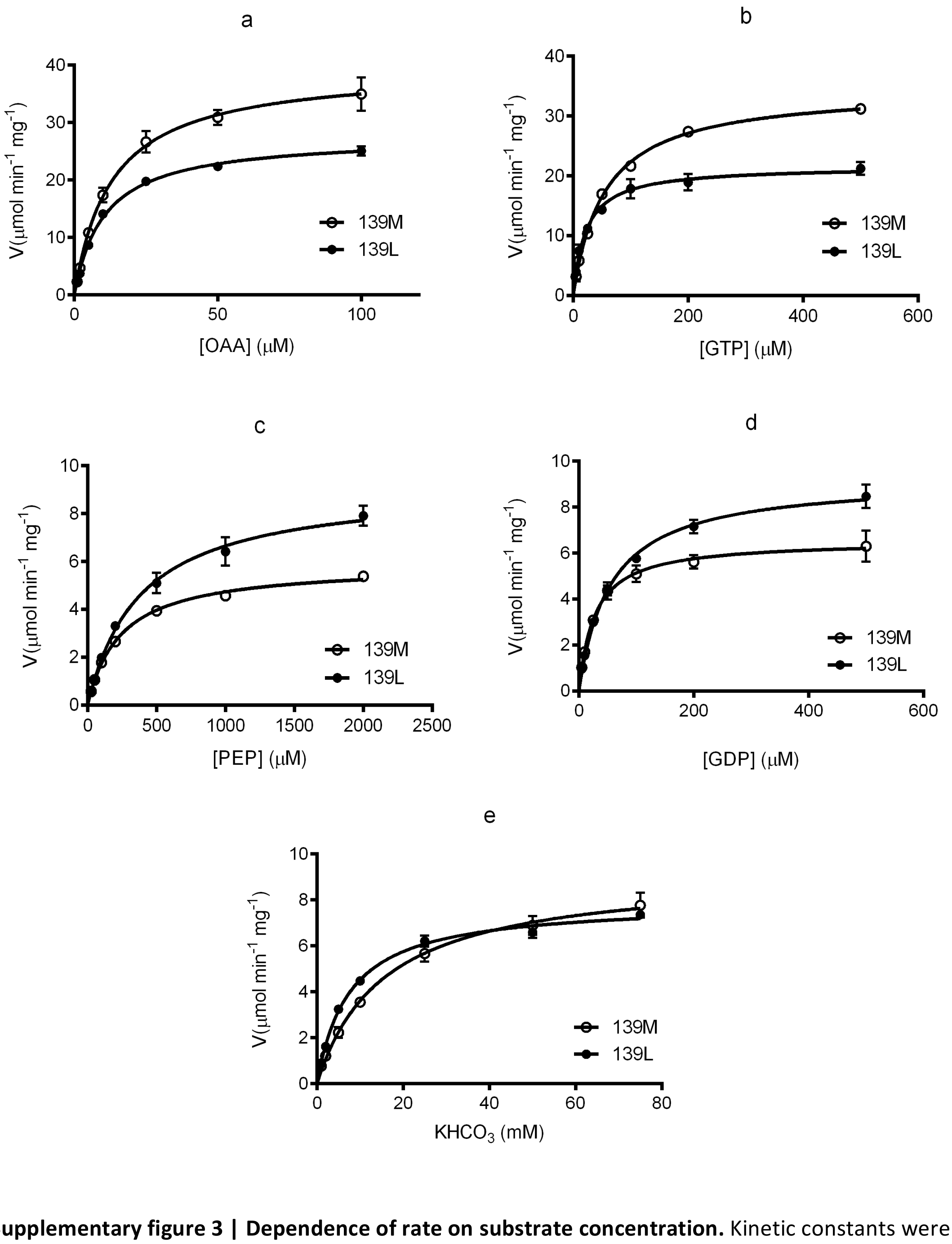

Supplementary figure 3 | Dependence of rate on substrate concentration. Kinetic constants were calculated from these data using EnzFitter, a non-linear regression software for enzyme kinetics. The main substrates of the Pck1 reaction **(a)** oxaloacetic acid (OAA), **(b)** GTP, (**c**) phosphoenolpyruvate (PEP), **(d)** GDP and **(e)** $KHCO_3$ were studied.

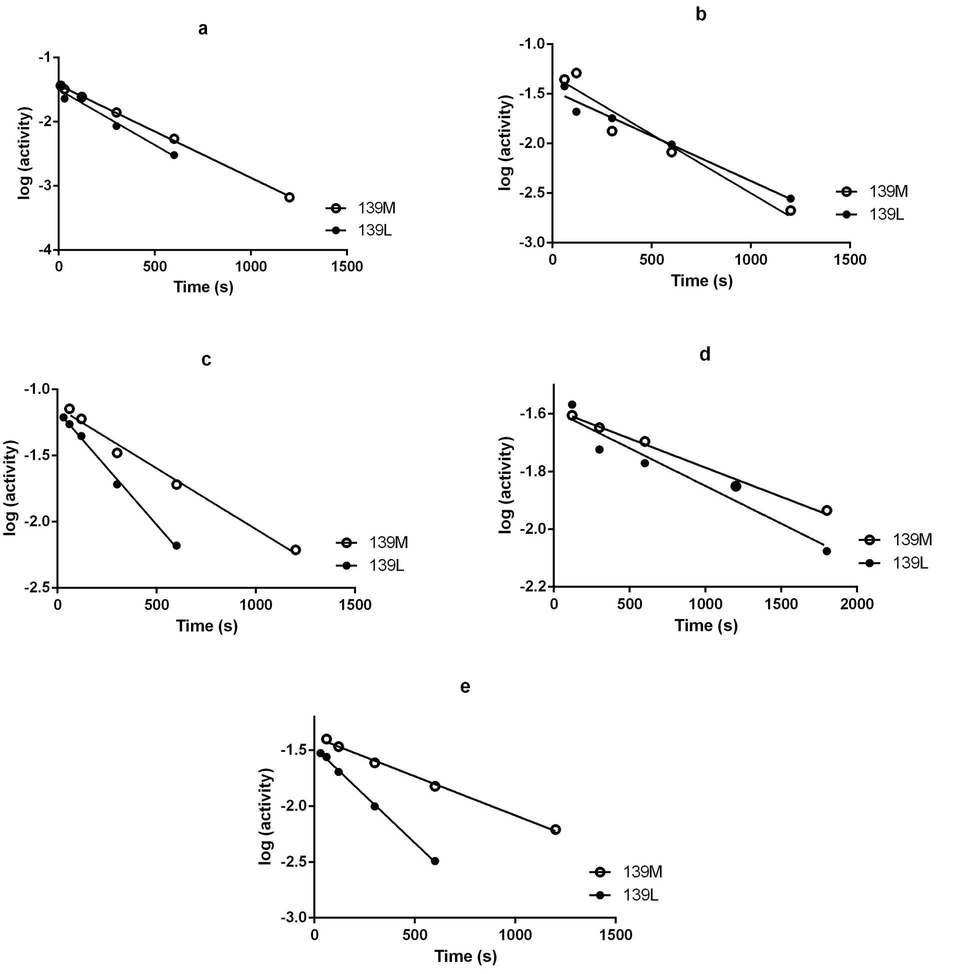

Supplementary figure 4 | Semilog plots for D_t calculations. Enzyme aliquots were heated at 50ºC constant temperature for different times. Then, they were immediately cooled and assayed for residual activity. The figure shows representative experiments performed in **(a)** the absence of substrates or in the presence of **(b)** OAA, **(c)** GTP, **(d)** PEP or **(e)** GDP.

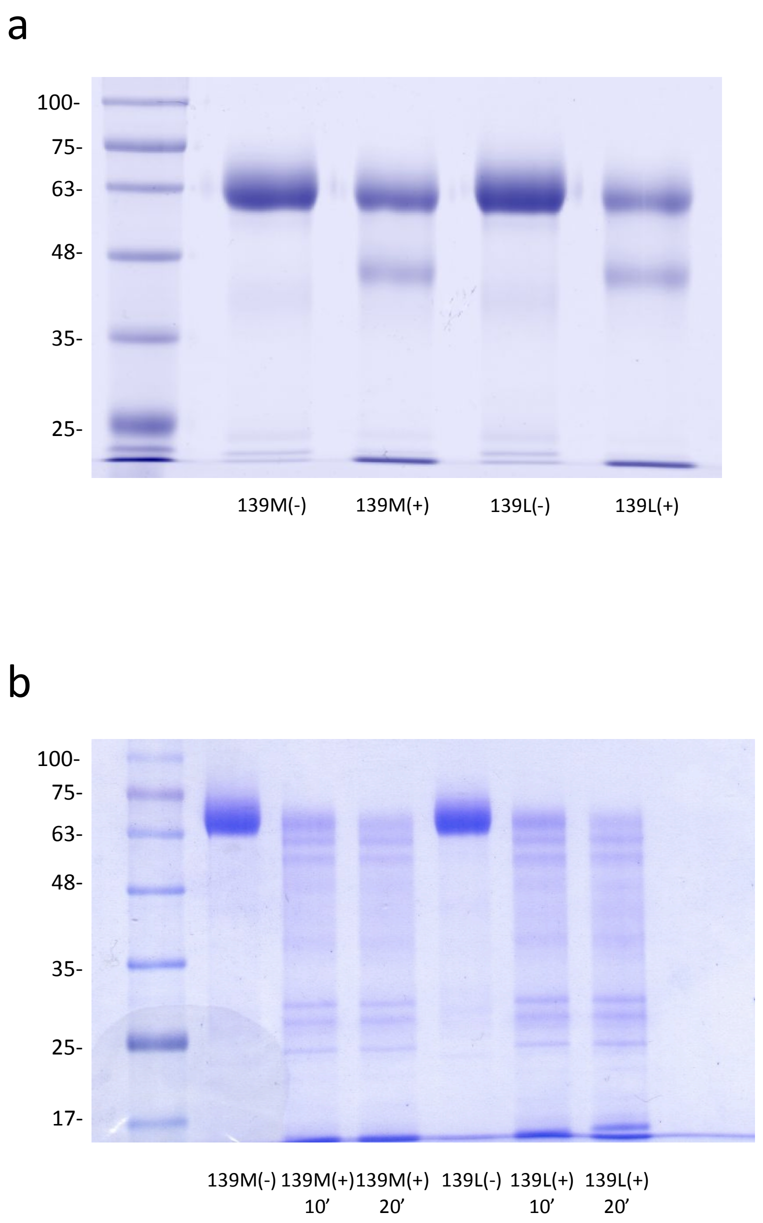

Supplementary figure 5 | Trypsin and proteinase K sensitivity of Pck1 p.139Met and Pck1 p.139Leu. (a) Trypsin sensitivity was assayed preparing 10 µg/mL trypsin and mixing 2 µL of this solution with 10 µL of Pck1 (0.5 mg/mL). Incubation took place at 37ºC for 18h. For the **(b)** proteinase K sensitivity assay, protease was prepared at 2 µg/mL. Pck1 and proteinase K were mixed as described for trypsin sensitivity but incubated at 37ºC for 10 or 20 minutes. The reaction was stopped by adding 2x SDS-loading buffer and boiling for 5 minutes. No differences were observed between Pck1 p.139Met and Pck1 p.139Leu hydrolysis band pattern using these proteases.
